# Pial Vessel-Associated Microglia/Macrophages Increase in Female Dahl-SS/Jr Rats Independent of Pregnancy History

**DOI:** 10.3390/ijms23063384

**Published:** 2022-03-21

**Authors:** Junie P. Warrington, Qingmei Shao, Ahsia M. Clayton, Kenji J. Maeda, Ashtin G. Beckett, Michael R. Garrett, Jennifer M. Sasser

**Affiliations:** 1 Department of Neurology, University of Mississippi Medical Center, Jackson, MS 39216, USA; qshao2@umc.edu (Q.S.); ahsia_clayton@brown.edu (A.M.C.); abeckett@umc.edu (A.G.B.); 2 Department of Neurobiology & Anatomical Sciences, Neuro Institute, University of Mississippi Medical Center, Jackson, MS 39216, USA; 3 Department of Pharmacology & Toxicology, University of Mississippi Medical Center, Jackson, MS 39216, USA; kjmaeda42@gmail.com (K.J.M.); mrgarrett@umc.edu (M.R.G.); jennysasser@gmail.com (J.M.S.)

**Keywords:** meninges, border-associated cells, vascular-associated microglia, perivascular macrophages, hypertension, myeloid cells, Iba1

## Abstract

As the resident immune cells of the central nervous system, microglia have a wide range of functions such as surveillance, phagocytosis, and signaling through production of chemokines and cytokines. Recent studies have identified and characterized macrophages residing at the meninges, a series of layers surrounding the brain and spinal cord. While perivascular microglia within the brain parenchyma increase following chronic hypertension, there are no reports of changes at the meninges, and specifically, associated with the pial vasculature. Thus, we used female Sprague Dawley and Dahl salt-sensitive (SS/Jr) rat brains, stained for ionized calcium-binding adapter molecule (Iba1), and characterized microglia/macrophages associated with pial vessels in the posterior brain. Results indicate that Iba1^+^ pial vessel-associated microglia (PVAM) completely surrounded the vessels in brains from the Dahl-SS/Jr rats. PVAM density was significantly higher and distance between PVAMs lower in Dahl-SS/Jr compared to the Sprague Dawley rat brains. Pregnancy history did not affect these findings. While the functional role of these cells are not known, we contextualize our novel findings with that of other studies assessing or characterizing myeloid cells at the borders of the CNS (meninges and choroid plexus) and perivascular macrophages and propose their possible origin in the Dahl-SS/Jr model of chronic hypertension.

## 1. Introduction

The meninges comprise three distinct layers surrounding the brain and spinal cord: the dura mater, the arachnoid mater, and the pia mater. Each layer of the meninges has its own network of blood vessels and an intricate immune system. An excellent review of the meninges and its immune characteristics can be found here [1]. Pial vessels have a functional blood–brain barrier (BBB) and reside in the subarachnoid space, where cerebrospinal fluid (CSF) is produced and circulates. These vessels are therefore uniquely positioned to be modulated by CSF constituents. Structurally, pial arteries are similar to arteries found in other vascular beds: endothelial cells line the lumen and are connected by tight junctions. Smooth muscle cells surround the endothelial cell layer and provide most of the contractile function of the arteries. The structure and function of pial arteries have been described and have been extensively investigated (reviewed in [2]). 

Hypertension, especially chronic hypertension, induces vascular remodeling such that the vessel wall thickens, and lumen diameter decreases [3,4]. A seminal study showed that hypertension induces migration of microglia towards parenchymal vessels, and provided evidence that microglia proliferation and migration to the perivascular space preceded vascular dysfunction [5]. Perivascular cells have been described in studies from the 1980s [6,7]. Since then, more studies have emphasized the diversity and complexity of the perivascular cells. A comprehensive review of the cerebrovascular-associated immune cells has helped to distinguish between perivascular macrophages and vascular-associated microglia based on differences in cell markers and locations [8]. While the study by Koizumi et al. [5] demonstrated a deleterious role for perivascular microglia in the setting of hypertension, it is not known whether similar events occur with pial vessels in rats with chronic hypertension. Additionally, whether pregnancy history influences density of pial vessel-associated microglia (PVAM) is not known.

The Dahl salt-sensitive (SS/Jr) female rat, when pregnant, displays features similar to women with superimposed preeclampsia [9], a hypertensive pregnancy disorder, where women with chronic hypertension develop preeclampsia symptoms during pregnancy [10]. Importantly, the postpartum Dahl-SS/Jr rats exhibit renal injury compared to sexually-naïve Dahl-SS/Jr female rats despite having similar blood pressures [11]. A recent study from our group demonstrated that during pregnancy, these rats have increased BBB permeability through disruption of the capillary endothelial tight junctions [12]. Changes in the structure of the cerebral vessels in the postpartum period have not yet been investigated.

Thus, in this study, we characterized changes in perivascular microglia/macrophages associated with pial vessels in female rats with chronic hypertension versus control Sprague Dawley rats that were never pregnant or had undergone two pregnancies. We also investigated whether the PVAMs expressed markers suggestive of an antigen-presenting phenotype.

## 2. Results

### 2.1. General Characteristics of Rats

There were no differences in body weight, hematocrit, or blood pressure between female Sprague Dawley and Dahl-SS/Jr rats regardless of pregnancy history (Table 1). Not all rats had measured data for all endpoints as indicated in the table. Quantification of vascular parameters such as outer vascular perimeter, media thickness, and number of PVAMs is provided in Table 1. There was a main effect of rat strain on the outer perimeter [F(1, 33) = 5.928; *p* = 0.020], where Dahl-SS rats had a longer perimeter than the SD rats (Table 1). There was no effect of pregnancy history on the outer perimeter [F(1, 33) = 0.468; *p* = 0.499]. In terms of wall thickness, there was no effect of strain or pregnancy history (Table 1). There was a significant effect of rat strain on the number of PVAMs [F(1, 33) = 26.65; *p* < 0.001], with Dahl-SS rats having a higher number of PVAMs (Table 1). 

### 2.2. Perivascular Microglia/Macrophages Associate with Pial Vessels

**Figure 1:**
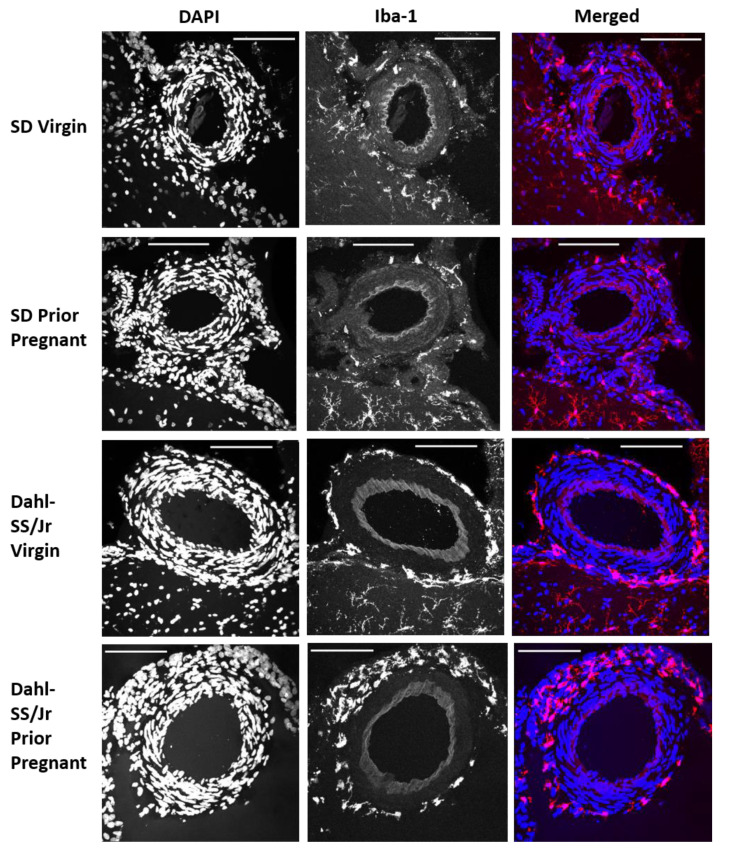
Representative maximum projection confocal images of pial vessels from each group. Brain sections were stained for Iba1 and co-stained using DAPI. Pial vessels from SD virgin, SD prior pregnant, Dahl-SS/Jr virgin, and Dahl-SS/Jr prior pregnant rats are shown. Microglia/macrophages (Iba1+) are red and nuclei (DAPI+) are blue. Scale bar = 50 µm.

Representative images of PVAMs surrounding pial vessels from each group are shown in Figure 1. In the Dahl-SS/Jr group, microglia almost completely surrounded the pial blood vessel. PVAMs were more sparse and infrequent in the SD brains. 

### 2.3. Dahl-SS/Jr Rats Have Higher Density of and Decreased Distance between PVAMs 

**Figure 2:**
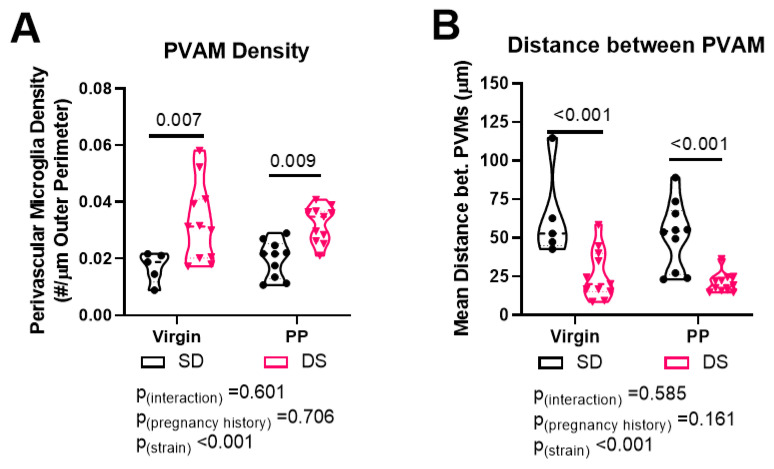
Pial vessel coverage by PVAMs is higher in Dahl-SS/Jr females. Violin plots showing (**A**) PVAM density and (**B**) distance between PVAM in brains from virgin and prior pregnant Dahl-SS/Jr rats compared to SD rats. Individual data points per rat are shown.

There was a main effect of rat strain on microglia/macrophage density [F(1, 33) = 19.12, *p* < 0.001], with Dahl-SS/Jr rats having a higher density of PVAM compared to SD rats. There was no effect of pregnancy history on PVAM density. Within the virgin groups, PVAM density was higher in the Dahl-SS rats (0.031 ± 0.014 vs. 0.0170 ± 0.005 PVAM/µm; *p* = 0.007). Prior pregnant Dahl-SS rats had higher PVAM density compared to prior pregnant SD rats (0.032 ± 0.006 vs. 0.020 ± 0.006 PVAM/µm; *p* = 0.009), as shown in Figure 2A.

We then measured the distance between each microglia/macrophage process surrounding the blood vessels and calculated the mean distance between microglia/macrophage for each rat. There was a main effect of rat strain on distance between PVAM, with Dahl-SS/Jr rats having reduced distance [F(1, 33) = 30.08, *p* < 0.001]. There was no effect of pregnancy history on the distance between PVAMs. Pairwise comparison revealed significant decreases in distance between PVAMs in the Dahl-SS/Jr rats in both the virgins (27.96 ± 16.07 vs. 64.02 ± 29.29 µm, *p* < 0.001) and prior pregnant (20.74 ± 6.38 vs. 51.61 ± 21.86 µm, *p* < 0.001) groups, as shown in Figure 2B. Post hoc power analysis revealed that power to detect a strain difference was 1, for pregnancy history 0.138 or 1, and for interaction 0.376 or 0.435. Thus, our study was sufficiently powered.

### 2.4. Pial Perivascular Microglia/Macrophages Are Distinct from the Glia Limitans

**Figure 3:**
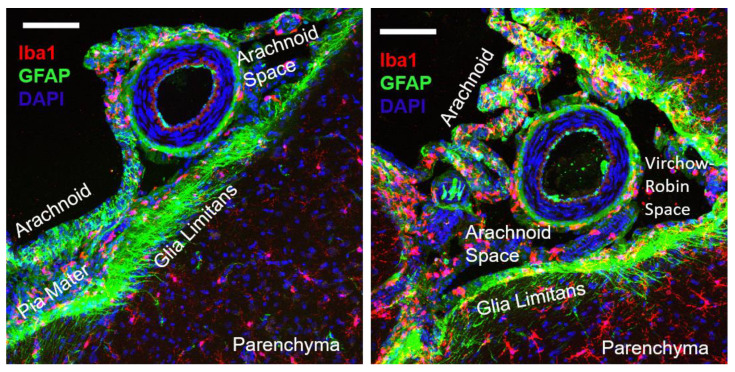
Representative images from a Dahl-SS/Jr prior pregnant female showing the distinct location of perivascular microglia relative to the glia limitans. Iba1+ cells could be seen along the pia mater, arachnoid, parenchyma, and surrounding the pial vessel. The glia limitans can be visualized based on GFAP expression. Red = Iba1. Green = GFAP. Blue = DAPI. Scale bar = 50 µm.

To determine the relative location of the perivascular microglia/macrophages, we co-stained some sections with glial fibrillary acidic protein (GFAP) and, as shown in Figure 3, PVAMs were distinct from the glia limitans. Several Iba1+ cells were visible in the arachnoid and pia mater, but these cells appeared distinct from those that were surrounding the pial vessels. 

### 2.5. Some Perivascular Microglia/Macrophages Co-Expressed Major Histocompatibility Complex II (MHC-II)

**Figure 4:**
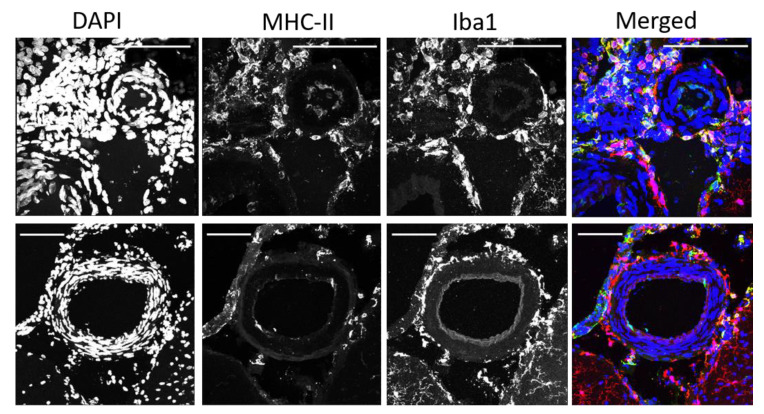
Representative images from a Dahl-SS/Jr prior pregnant female showing MHC-II staining in PVAMs. Some PVAM were double positive for Iba1 and MHC-II. Upper Panel: Pial arteriole surrounded by Iba1+/MHC-II+ PVAM. Lower Panel: Pial vessel with PVAMs of varying distances surrounding it with associated arachnoid layer at the cortical surface. Red = Iba1. Green = MHC-II. Blue = DAPI. Scale bar = 100 µm.

We co-stained a subset of brain sections with major histocompatibility complex (MHC-II) antibody and found that some of the PVAMs co-expressed MHC-II, demonstrating that some cells are antigen-presenting cells (Figure 4). The top panels of Figure 4 show a pial arteriole that is almost completely surrounded by Iba-1+ cells. The vast majority of the cells also expressed MHC-II. In the bottom panel, a subset of Iba1^+^/MHC-II^+^ cells are seen associated with the arachnoid layer and associated with the pial vessel. 

### 2.6. The Density of MHCII-Positive PVAM Increased in Dahl-SS/Jr Female Rats

**Figure 5:**
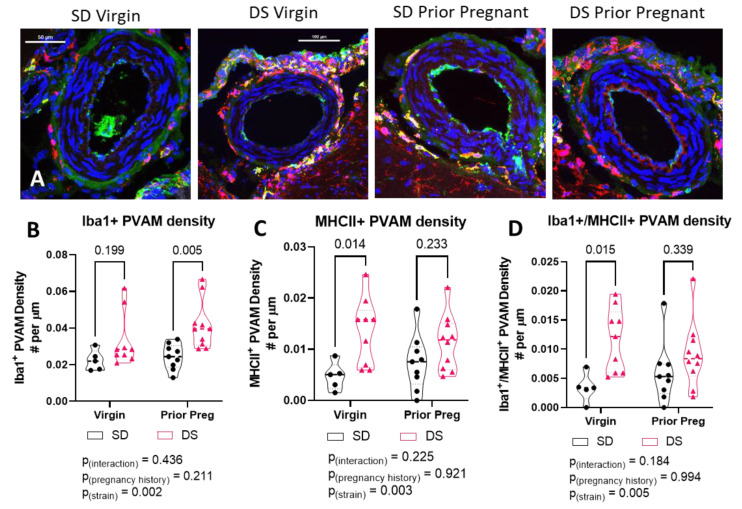
Representative images from each group showing Iba1 and MHC-II staining in PVAMs (**A**). Images were then analyzed. There was only a main effect of strain on density of PVAMs that were Iba1+ (**B**), MHCII+ (**C**) or Iba1+/MHCII+ (**D**). Red = Iba1, Green = MHC-II, Blue = DAPI. Scale bar = 100µm. SD—Sprague Dawley, DS—Dahl-SS/Jr.

We quantified the number of PVAMs associated with pial vessels in rat brain slices using sections that were 3 sections caudal to the ones quantified in Figure 2. There was a significant strain effect on Iba1+ PVAM density which was driven mainly by the prior pregnant female rats (Figure 5B). MHCII+ PVAMs increased in the Dahl-SS/Jr females but occurred primarily in the virgin group (Figure 5C). Lastly, double-positive PVAM density increased in the Dahl-SS/Jr female rats but was driven by the virgin, never pregnant group (Figure 5D). Post hoc power analysis revealed that this study was sufficiently powered, with power of >0.9 for interaction, 1 for strain, and 0.05 to 1 for pregnancy history. 

### 2.7. The Morphology of the PVAMs Differed in Different Brains

**Figure 6:**
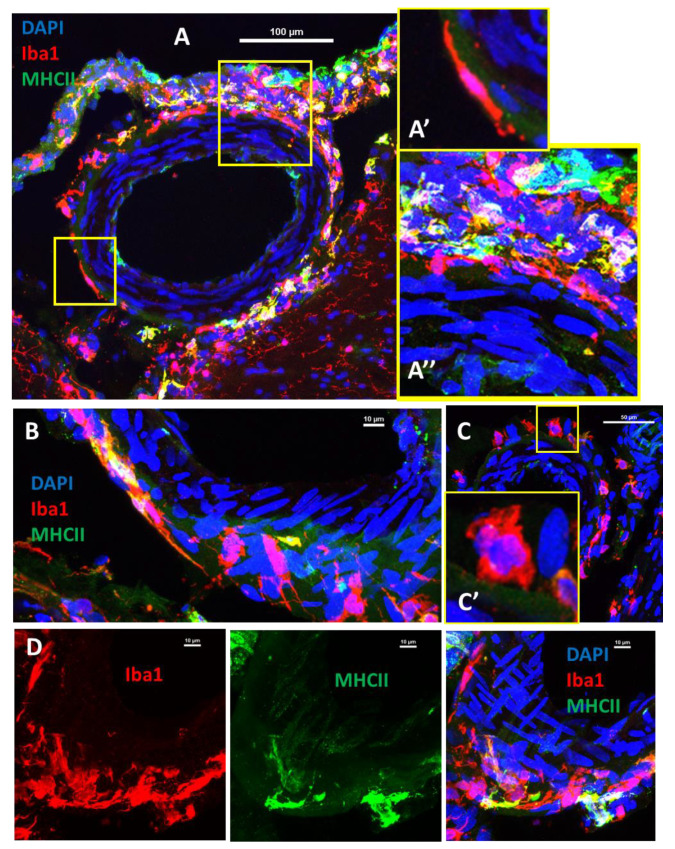
Representative images showing different PVAM morphology. Some PVAMs are elongated around the circumference of the pial vessel’s cross-section (**A′**,**A″**) while others appeared to run perpendicular to the smooth muscle cells (**B**). Other cells appeared more amoeboid and round with greater distance between them (**C**,**C′**). (**D**) Additional PVAMs in close proximity to each other around the pial vessels. Red = Iba1, Green = MHC-II, Blue = DAPI. Scale bar in A = 100 µm and B–D = 10 µm. SD—Sprague Dawley, DS—Dahl-SS/Jr.

We captured higher-magnification images of the PVAMs and found different morphologies, some of which are pictured in Figure 6. PVAMs appeared elongated in some of the blood vessels, others were more rounded, while others appeared to be positioned perpendicular to the smooth muscle cell layers. Thus, PVAMs have varying morphological presentations.

## 3. Discussion

The Circle of Willis is supplied by the carotid and vertebral arteries and is located on the inferior surface of the brain. The anterior, middle, and posterior cerebral arteries branch from the Circle of Willis and serve as the primary blood supply to the cerebrum. During development, microglia originate from the yolk sac, invade the brain from the pia, migrate along blood vessels and radial glia, proliferate, and transform to a ramified morphology at their destination [13]. In adulthood, the vast majority of microglia are located within the parenchyma, although microglia-like cells have been found and characterized at the lepto-meninges or near the ventricles [14,15]. In the current study, we present the first report of a scenario where pial vessels are surrounded by microglia/macrophages (Iba1^+^ cells), forming almost a complete additional vascular barrier. We report that PVAM density is increased and the distance between PVAM is lower in the Dahl-SS/Jr rat strain, a hypertensive model, with mean arterial blood pressure averaging 157mmHg. We also show that the PVAMs are distinct from the glia limitans, and some of the PVAMs are antigen presenting cells and co-express MHC-II with Dahl-SS/Jr groups having higher densities of MHCII^+^ PVAMs. Our analysis revealed no effect of pregnancy history on PVAM density around pial vessels.

The distinction between perivascular microglia and macrophages is controversial and some groups have proposed that perivascular cells are distinct from pericytes, microglia, and macrophages [16]. In the current study, we identified the perivascular cells as microglia because of the high expression of Iba1, a microglia marker [17]. Recent studies have begun to identify and characterize different macrophage and immune cell populations associated with the brain borders. A summary of studies assessing perivascular cells at the CNS borders is provided in Table 2. Using modern techniques such as single-cell RNA sequencing, investigators have shown that there are different populations of macrophages and microglia at the brain borders with distinct profiles that are unlike parenchymal microglia [15]. However, the studies did not differentiate between perivascular cells and meningeal cells not associated with the vasculature.

Microglia within the parenchyma function to actively surveil the microenvironment and respond to injury by phagocytizing debris and dead cells [25]. Under resting conditions, microglia use filopodia, located at the tip of large processes, to surveil the microenvironment [26]. In the current study, we did not determine whether filopodia are present on the microglia/macrophage processes surrounding the pial vessels. Nonetheless, it is possible that filopodia are present on the PVAM and participate in surveilling the CSF, considering that the Iba1^+^ perivascular cells in the current study are aptly positioned to do so. Evidence for the phagocytic nature of perivascular cells has been reported previously. For example, injection of Indian ink into the subarachnoid space resulted in drainage along paravascular pathways into nasal lymphatics and cervical lymph nodes. The authors showed that perivascular cells rapidly and efficiently ingested the ink [27] and that perivascular cells, defined by their expression of ED2, acted as scavengers to Indian ink [16]. Additionally, perivascular microglia have been described as “constitutively phagocytic” by Serrats et al. [28]. Moreover, fluorescent granular perithelial cells from the pia have been shown to occupy the perivascular spaces, have pinocytosis ability, and function to scavenge waste products [29].

In addition to surveilling the CSF, because of their orientation around the blood vessels, PVAM may also scavenge components that leak from the blood and process antigens presented by infiltrating lymphocytes following vascular injury and play an active role in phagocytosing degenerating axons within the parenchyma [30,31]. Together, these studies support the hypothesis that PVAM function to phagocytize debris and other substances that may circulate in the CSF or leak from damaged pial vessels and continuously surveil the subarachnoid space.

Some of the perivascular cells identified in the current study were MHC-II^+^, suggesting that they are capable of presenting antigens to lymphocytes. An early study showed that perivascular microglia arise from the bone marrow and act as antigen-presenting cells around the vessels (arterioles and venules) [6]. ED-2-positive perivascular cells, distinct from pericytes and parenchymal microglia, have also been described in rat brains [7].

The closeness of the perivascular cells to each other (~25 µm apart in the Dahl-SS/Jr rats) around the blood vessels hint to a potential function of strengthening the BBB. There is evidence that chronic hypertension induces vascular remodeling of the pial vessels to compensate for the increased blood pressure [4,32]. Using ex vivo studies, inward remodeling of the pial vessels (smaller lumen diameter and thicker vessel wall) has been reported [32]. Moreover, increases in blood flow and transmission of pressure to the micro-vessels causes increased vascular leakage. Our observations of closely arranged perivascular microglial cells suggest that they may represent compensatory changes to protect the BBB. This hypothesis needs to be tested by depleting PVAMs at the CNS borders and measuring BBB permeability.

Evidence for the alternative hypothesis also exists. One study showed that following the acute phase of ischemia/reperfusion, border-associated macrophages increased the permeability of pial and cortical vessels [23]. Most of the work in hypertension has focused on perivascular macrophages rather than PVAMs. In those studies, increased perivascular macrophages was shown to mediate the increase in BBB permeability and contribute to cognitive impairment in response to angiotensin II-induced hypertension [19]. The authors went on to show that perivascular macrophage depletion or selective deletion of CD36 or Nox2 specifically from perivascular macrophages protected mice from vascular damage induced by amyloid beta [20]. These studies all focused on parenchymal vessels and did not assess pial vessels or PVAMs. Microglia and macrophages are known to produce and secrete cytokines and chemokines [33]. It is possible that the Iba1^+^ PVAMs are contributing to further injury by secreting pro-inflammatory cytokines into the CSF. This hypothesis will be investigated in future studies.

One of our unanswered questions is where the perivascular cells come from. One potential source could be circulating blood monocytes. There is evidence that perivascular macrophages arise from circulating blood monocytes. Bone marrow transplant studies have shown that following depletion of BM cells, transplanted cells migrate to the perivascular spaces throughout the brain [34]. The study also showed that the turnover rate of these perivascular cells was very high with almost all of the Iba1^+^ perivascular cells at 14 weeks post-transplantation being from the donor. In contrast, using bone marrow transplant, Albini et al. demonstrated that donor-derived microglia were not detected in brain or retina samples until after 52 weeks post-transplantation [35]. It is therefore possible that the PVAMs observed in the rat brains represent cells produced in bone marrow that circulated within blood vessels and extravasate to their perivascular location surrounding the pial vessels.

Another potential source is through proliferation of resident microglia/macrophages. Indeed, microglia populations are maintained through active proliferation during adulthood [36]. Several studies assessing proliferation of parenchymal microglia were discussed and summarized in [14]. Evidence of microglia proliferation in adult rodents was provided by a seminal study in DOCA-treated rats where hypertension induced perivascular location of microglia and proliferation of perivascular microglia, before vascular damage was observed [5]. We have not assessed whether the PVAMs are actively proliferating or whether vascular damage occurs from the presence of these cells.

Another potential source of the pial PVAMs could be from the CSF that bathes the blood vessels. A recent paper showed that a unique subset of microglia-like cells were found in HIV^+^ patients with viral counts controlled using anti-viral medication [22]. In that study, single-cell RNA sequencing was used to characterize the transcriptome of CSF and blood cells from HIV^+^ and control participants. Results indicated that the CSF contained myeloid cells with similar characteristics as microglia. HIV^+^ participants had more of these myeloid transcripts in the CSF than control participants and higher transcripts compared to blood cells. Taken together, this study demonstrated that microglia-like cells can be detected in the CSF and could present a pool of cells that could integrate near pial vessels that are damaged. Studies are required to assess whether the PVAM described here are similar to those circulating in the CSF. We hypothesize that based on the location of the PVAMs in the arachnoid space where CSF circulates, that CSF could be a potential source of the PVAMs.

Our current findings indicate that there was no effect of pregnancy history on the density of PVAMs in rat brains and that regardless of pregnancy history, chronic hypertension, a feature of the Dahl-SS/Jr strain, contributed to increased PVAM density. To our knowledge, this finding suggests that the PVAMs may present a compensatory mechanism that develops in response to chronic hypertension as occurs in the Dahl-SS/Jr strain. Several studies indicate that hypertensive disorders of pregnancy increase the risk of cerebrovascular complications such as stroke both during pregnancy and the postpartum period. For example, a nationwide study in Taiwan showed that women with chronic hypertension and superimposed preeclampsia during pregnancy had a significant risk of stroke in the postpartum period (up to 17 years after index pregnancy) [37]. Moreover, women with a history of PE have a significantly higher risk of developing vascular dementia; and this risk is even higher if the pregnancy resulted in a small-for-gestational-age baby [38]. Thus, we predicted that the prior pregnant Dahl-SS/Jr rats would have different pial vascular structure compared to littermates that were never pregnant. Our findings of a lack of significant pregnancy effect indicate that the increase in PVAM density is driven primarily by chronic hypertension or could be a strain effect. It should be noted that the lowest power values were obtained for the pregnancy history factor, with a large range obtained from the different endpoints being considered. Further studies would benefit from increasing the number of animals in the Sprague Dawley virgin group to a comparable sample size as the other groups. Further studies will need to be performed to determine if treating hypertension during pregnancy and potentially postpartum will prevent this vascular phenotype in the Dahl-SS female rats.

## 4. Materials and Methods

### 4.1. Animals

Female Sprague Dawley (SD) and Dahl-SS/Jr rats, had ad libitum access to a low-salt diet (0.3% salt) and water and were housed at the University of Mississippi Medical Center (UMMC) Lab Animal Facilities. Rats were kept on a 12 h light/12 h dark cycle in a humidity and temperature-controlled environment. At 3 months of age, female rats (*n* = 10 SD and *n* = 11 Dahl-SS/Jr) were mated with males of the same strain and age and allowed to deliver their pups. Dams were subjected to a second pregnancy at approximately 4.5 months of age and allowed to deliver again. At 3.5 months following the second delivery, rats (weighing 243–352 g) were anesthetized using isoflurane and were perfused using 1X phosphate-buffered saline. Tissues were then harvested. Female rats that were never mated (*n* = 5 SD and *n* = 11 Dahl-SS/Jr) were used as controls. All animal procedures were approved by the Institutional Animal Care and Use Committee at UMMC. 

### 4.2. Immunofluorescence Staining

Rat brains were post-fixed in 4% paraformaldehyde overnight followed by 30% sucrose at 4 °C. Brains were then embedded in Cryogel, frozen, and then stored at −80 °C until further processing for immunofluorescence staining, as previously described [39]. Coronal slices (20 μm thick) were cut, transferred to slides, and kept at −20 °C until staining. Frozen sections (2 sections per slide) were warmed to room temperature for 15 min. Sections were blocked with 10% normal donkey serum for 2 h after washing with 1X phosphate-buffered saline (PBST) for 5 min. Sections were then incubated with a primary antibody (1:500 Rabbit anti-Iba1; Wako, Code No: 019-19741) or mixture of two antibodies—1:500 Rabbit anti-Iba1 and 1:100 Mouse anti-MHC II (abcam, Cat. No: ab23990) OR 1:500 Rabbit anti-Iba1 and 1:1000 Mouse anti-GFAP (abcam, Cat. No: ab23990)—to cover the sections, and then incubated overnight at 4 °C. The next day, sections were washed with 1X PBST, 3 times 5 min, followed by incubation with a secondary antibody, 1:600 Donkey anti-Rabbit TRITC (Jackson Lab. Code No: 711-025-152), or mixture of two secondary antibodies—1:600 Donkey anti-Rabbit TRITC and 1:1000 Donkey anti-Mouse Alexa Fluor 488 (Jackson Lab, Code No: 715-545-150)—to cover the sections and incubated for 1 h at room temperature. After washing three times with 1XPBST, slides were covered with Anti-fade Mounting Medium with DAPI (Vector Lab. Cat. No: H-1500) and cover-slipped.

### 4.3. Confocal Imaging and Analysis

Z-stacks (512 × 512 pixels) of 1 µm steps were captured through the 20 µm brain sections (1 section per rat brain) using confocal microscopy. We scanned the edges of the brain and imaged the vessels at the inferior portion of the brain, towards the midline. These pial vessels are located in the region of the posterior cerebral arteries. Maximum projection images were used for analysis. Using the measurement tool in NIS-Elements (Nikon), the number of Iba1^+^ microglia immediately surrounding pial arteries were counted. The outer perimeter of the blood vessel was measured and PVAM density calculated by dividing the number of PVAM by the vessel perimeter (#/µm). The distance between each microglia/macrophage was measured and averaged to obtain the mean distance between microglia. 

### 4.4. Statistical Analysis

Two-way analysis of variance was used to determine the effect of strain and pregnancy on PVAM measures. Pairwise differences were calculated using Sidak’s multiple comparison post hoc test. Significance was established at *p* < 0.05 threshold. Statistical analyses were performed and graphs created using GraphPad Prism (version 8.3.0). Post hoc power analysis was performed using G*Power (ver 3.1.9.7) using the F values calculated from GraphPad Prism, and alpha set to 0.05.

## 5. Conclusions

Perivascular cells may have dual roles, where they participate in surveillance and phagocytosis, restoring homeostasis or they could contribute to pathophysiology through chronic secretion of pro-inflammatory cytokines and increased BBB permeability. As discussed, there are several different names and markers used to identify perivascular cells. Much of the literature focuses on perivascular macrophages and fewer assess PVAM. Others have classified these cells into their own category of perivascular cells. Recent transcriptomic profiling of border-associated cells has shed some light on the true diversity of cells at the borders of the CNS. Even with advances in identifying different populations of cells based on their transcriptional profile, the methodologies are limited by the starting material used to isolate the cells. Florescence-activated cell sorting methods limit us to a specific starting population of cells based on their surface markers. In our current analysis, the identified perivascular cells are likely microglia since they have a high expression of Iba1 and some of them are antigen-presenting cells. Future studies will assess whether these PVAMs are present throughout development, in males, in different animal species, or other models of chronic hypertension. 

## Figures and Tables

**Table 1:** General and vascular characteristics of rats.

Measurement	SD Virgin	SD Prior Pregnant	Dahl-SS Virgin	Dahl-SS Prior Pregnant
Body Weight (g)	291 ± 10 (5)	294 ± 6 (5)	254 ± 12 (3)	307 ± 39 (3)
Hematocrit (%)	Not assessed	44 ± 3 (5)	49 ± 5 (9)	48 ± 3 (9)
Mean Arterial Pressure (mmHg)	Not assessed	144 ± 22 (4)	157 ± 14 (8)	156 ± 11 (7)
*Pial Vessel Parameters*				
Outer Perimeter (µm)	587± 107 (5)	527 ± 109 (10)	622 ± 122 (11)	769 ± 253 (11) ‡
Wall Thickness (µm)	49 ± 15 (5)	43 ± 6 (10)	45 ± 6 (11)	48 ± 5 (11)
No. of PVAM	10 ± 3 (5)	11 ± 5 (10)	19 ± 9 (11) ¥	24 ± 6 (11) ‡

Data represent the mean ± SD. ‡ *p* ≤ 0.005 vs. SD prior pregnant group; ¥ *p* ≤ 0.01 vs. SD virgin. SD—Sprague Dawley; PVAM—pial vessel-associated microglia. Numbers in parentheses represent sample size for each measurement.

**Table 2:** Characteristics of different perivascular cells.

Cell Type	Markers Used	Location	Method	Main Findings	Ref
Perivascular Cells	ED2	Parenchyma	Rotring Indian Ink + Light Microscopy + IHC + EM	ED2^+^ cells in perivascular spaces took up the ink. No uptake by pericytes, macrophages, or microglia. Perivascular cells act as scavengers	[16]
Perivascular Macrophages	Iba1^low^	Subfornical Organ	IF, LPS injection	Iba1^+^ perivascular macrophages expressed IL-1β. May contribute to tolerance of endotoxin	[18]
Perivascular Macrophages	CD206 Lyve1 Iba1^low^	Parenchyma	IF, AngII infusion	Increased production of ROS, BBB leakage, cognitive impairments. Depletion of PVMs prevented these	[19]
Perivascular Macrophages	CD206 CD36	Parenchyma	IF	PVMs express Aβ receptor, CD36, and may contribute to the pathophysiology of Alzheimer’s disease	[20]
Perivascular Macrophages	CD163	MCA	IF	Depletion using Clodronate reduced perivascular macrophages in spontaneously hypertensive rats	[21]
Myeloid-2 Cells	TREM2, APOE	CSF	ScRNA-Seq	↑ Transcripts of myeloid cells in CSF from HIV^+^ participants compared to control and blood	[22]
Border-Associated Macrophages	CD163	Leptomenigeal spaces and Choroid plexus	scRNA-Seq Ischemia/reperfusion	Granulocyte recruitment, ↑ VEGF expression, ↑ pial and cortical vessel permeability, ↑ neurological dysfunction post-ischemia/reperfusion acutely	[23]
Border-Associated Macrophages	N/A	Dura mater, subdural meninges, choroid plexus	scRNA-Seq	Distinct tissue-specific transcriptional profiles. Unique microglial subset on apical surface of choroid plexus epithelium	[15]
Perivascular Macrophages/ Microglia	Cx3Cr1	Parenchyma	PLX5622, AngII	Depletion prevents AngII-induced short-term memory impairment	[24]

ED2—CD163 glycoprotein, IHC—immunohistochemistry, EM- electron microscopy, Iba1—ionized calcium-binding adaptor molecule 1, IF—immunofluorescence, LPS—lipopolysaccharide, Lyve1—lymphatic vessel endothelial hyaluronan receptor 1, AngII—angiotensin II, PVM—perivascular macrophage, ROS—reactive oxygen species, BBB—blood–brain barrier, MCA—middle cerebral arteries, TREM2—triggering receptor expressed on myeloid cells 2, APOE—apolipoprotein E, CSF—cerebrospinal fluid, VEGF—vascular endothelial growth factor, and Cx3Cr1—C-X3-C motif chemokine receptor 1.

## Data Availability

Data used in the generation of figures for this manuscript are available from the corresponding author upon request.
